# Differentially expressed transcripts of *Tetracapsuloides bryosalmonae* (Cnidaria) between carrier and dead-end hosts involved in key biological processes: novel insights from a coupled approach of FACS and RNA sequencing

**DOI:** 10.1186/s13567-023-01185-7

**Published:** 2023-06-26

**Authors:** Saloni Shivam, Reinhard Ertl, Veronika Sexl, Mansour El-Matbouli, Gokhlesh Kumar

**Affiliations:** 1 Division of Fish Health, University of Veterinary Medicine Vienna, Vienna, Austria; 2 Karwar Regional Station of Indian Council of Agricultural Research, Central Marine Fisheries Research Institute, Karwar, Karnataka India; 3 VetCore Facility for Research, University of Veterinary Medicine Vienna, Vienna, Austria; 4 Institute of Pharmacology and Toxicology, University of Veterinary Medicine Vienna, Vienna, Austria; 5 School of Biotechnology, Badr University in Cairo, Badr City, Cairo, Egypt

**Keywords:** Proliferative kidney disease, malacosporean endoparasite, differential transcript expression, brown trout, rainbow trout

## Abstract

*Tetracapsuloides bryosalmonae* is a malacosporean endoparasite that infects a wide range of salmonids and causes proliferative kidney disease (PKD). Brown trout serves as a carrier host whereas rainbow trout represents a dead-end host. We thus asked if the parasite adapts to the different hosts by changing molecular mechanisms. We used fluorescent activated cell sorting (FACS) to isolate parasites from the kidney of brown trout and rainbow trout following experimental infection with *T. bryosalmonae*. The sorted parasite cells were then subjected to RNA sequencing. By this approach, we identified 1120 parasite transcripts that were expressed differentially in parasites derived from brown trout and rainbow trout. We found elevated levels of transcripts related to cytoskeleton organisation, cell polarity, peptidyl-serine phosphorylation in parasites sorted from brown trout. In contrast, transcripts related to translation, ribonucleoprotein complex biogenesis and subunit organisation, non-membrane bounded organelle assembly, regulation of protein catabolic process and protein refolding were upregulated in rainbow trout-derived parasites. These findings show distinct molecular adaptations of parasites, which may underlie their distinct outcomes in the two hosts. Moreover, the identification of these differentially expressed transcripts may enable the identification of novel drug targets that may be exploited as treatment against *T. bryosalmonae*. We here also describe for the first time how FACS based isolation of *T. bryosalmonae* cells from infected kidney of fish fosters research and allows to define differentially expressed parasite transcripts in carrier and dead-end fish hosts.

**Supplementary Information:**

The online version contains supplementary material available at 10.1186/s13567-023-01185-7.

## Introduction

*Tetracapsuloides bryosalmonae*, the causative agent of proliferative kidney disease (PKD) is a malacosporean endoparasite of the phylum Cnidaria [1, 2]. It is a major parasite affecting freshwater salmonids in Europe and North America [3]. Similar to other myxozoans, *T. bryosalmonae* has a life cycle involving two hosts (vertebrate and invertebrate) [4]. The bryozoans serve as its primary host and act as reservoirs of *T. bryosalmonae* spores infective to their secondary hosts, the salmonid fish [4, 5]. The parasite undergoes a series of developmental alterations within both primary [6] and secondary hosts [7]. Spores released from infected bryozoan colonies infect salmonid fish mainly through their gills [8]; thereafter traverse through the blood vascular system to reach the main target organ kidney [9]. Within the kidney of fish host, the parasite transitions through extrasporogonic and sporogonic stages [7]. Subsequently, an intratubular development leads to the formation of spores [10], which leave the fish host via urine and are infective for the bryozoans [4, 8].

Although, numerous salmonids are susceptible to *T. bryosalmonae* presenting similar clinical signs and symptoms, currently only brown trout (*Salmo trutta*) and brook trout (*Salvelinus fontinalis*) are known to be carriers of the parasite [8]. Carrier fish are capable of releasing viable parasite spores, which are infective for the bryozoans. Rainbow trout, a dead-end host, upon infection exhibit similar clinical manifestations of the disease but do not shed spores infective for bryozoans, thereby terminating the parasite life cycle [8]. Interestingly, under similar environmental conditions, the appearance of clinical symptoms in both the fish host and also parasite development follows similar path. However, in contrast to brown trout, European strain of *T. bryosalmonae* never develops intra-luminal sporogonic stages in rainbow trout [7, 8].

Factors contributing to the different fate of *T. bryosalmonae* in rainbow trout and brown trout remain elusive. Apparently, the carrier state of brown trout suggests towards the existence of certain immune evasion strategies or manipulation of host pathways by the parasite. Likewise, the dead-end status of rainbow trout for this parasite might echo a successful and robust host response or the inefficient parasite survival tactic. Given the differential outcome of the parasite, comparative transcriptional profiling of *T. bryosalmonae* from rainbow trout and brown trout can be useful in gaining insights on the differential behavior of this parasite and elucidating important genes necessary for their survival and pathogenesis in the host. Knowledge of protective host immune responses and parasite biology could pave the way for developing successful PKD management measures including vaccines. Rational design of vaccines against any parasite including *T. bryosalmonae* could be based on either eliciting the host immune responses or targeting the parasite biology and/or virulence factors.

Transcriptomics has emerged as a powerful tool in advancing our understanding of host, parasite, and their interactions [11–14]. However, dominance of host transcripts has been highlighted as a major issue during dual RNA sequencing of parasite infected host samples, sometimes the parasite transcripts making a mere 0.1% [11]. Besides, a high probability of contamination from host exists. A possible solution would be to purify parasites from infected host tissues prior to RNA sequencing using techniques such as fluorescence-activated cell sorting (FACS). It is a high-throughput technique for selectively separating a desired cell type from a mixture of heterogeneous cell suspension with a high degree of purity [15]. Though this technique has emerged as a method of choice for isolating specific cell populations, it is also being used for the isolation and purification of parasites from a mixture of cell types [16].

In this study, we isolated *T. bryosalmonae* from the main target organ kidney of infected brown trout and rainbow trout using FACS and performed RNA sequencing of the sorted parasite cells to determine its differentially expressed transcripts (DETs) during the clinical phase of infection between the two fish hosts.

## Materials and methods

### Brown trout and rainbow trout sampling

Specific pathogen-free brown trout and rainbow trout (mean length 12 ± 2 cm) were purchased from a certified local hatchery and transported to our laboratory. The trout were acclimatized for a month in 1000 L tanks with continuous aeration and water flow. The temperature in tanks was maintained at 15 ± 1 °C. Fish were fed to satiation with commercial trout feed. Ten fish from each species were randomly sampled and their health status was ascertained by our routine diagnostic procedures. The examined fish were found to be negative for *T. bryosalmonae*, and other parasites and also for bacterial and viral pathogens.

**Figure 1:**
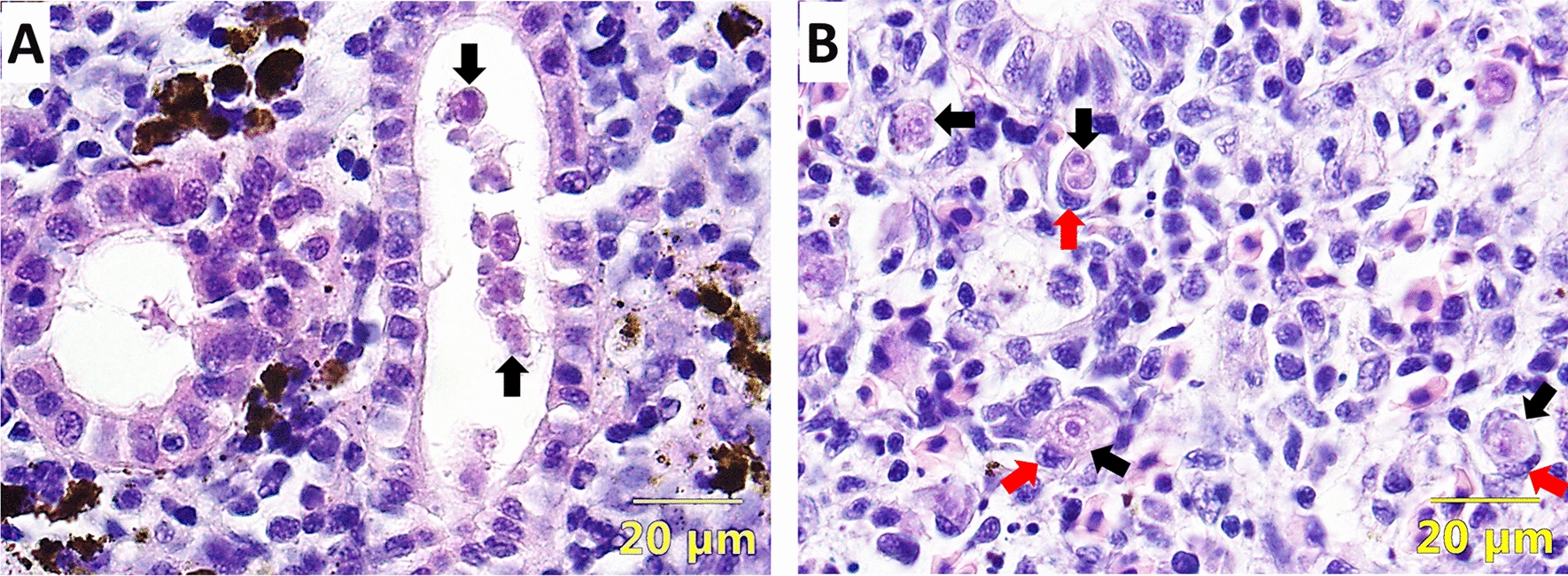
**Histological sections of infected kidneys.** **A** Brown trout kidney section shows intra-luminal sporogonic stages of *Tetracapsuloides bryosalmonae* (arrows), **B** rainbow trout kidney section shows interstitial pre-sporogonic stages (black arrows) of *T. bryosalmonae* associated with macrophages (red arrows) (H&E staining).

**Figure 2:**
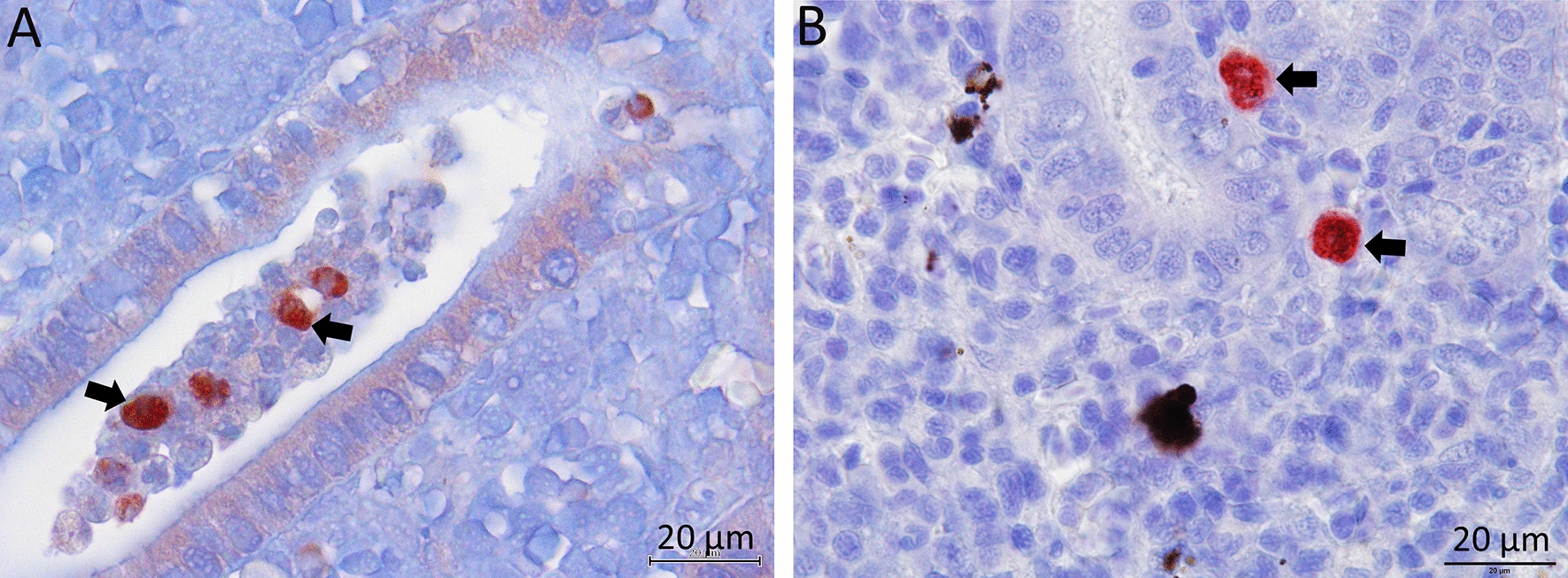
**Immunohistochemistry of infected kidneys.** **A** Brown trout kidney section shows intra-luminal sporogonic stages of *Tetracapsuloides bryosalmonae* (arrows), **B** rainbow trout kidney section shows interstitial pre-sporogonic stages of *T. bryosalmonae*. Parasite stages were visualized by immunohistochemistry using monoclonal antibody against *T. bryosalmonae* and counterstained with haematoxylin.

**Figure 3:**
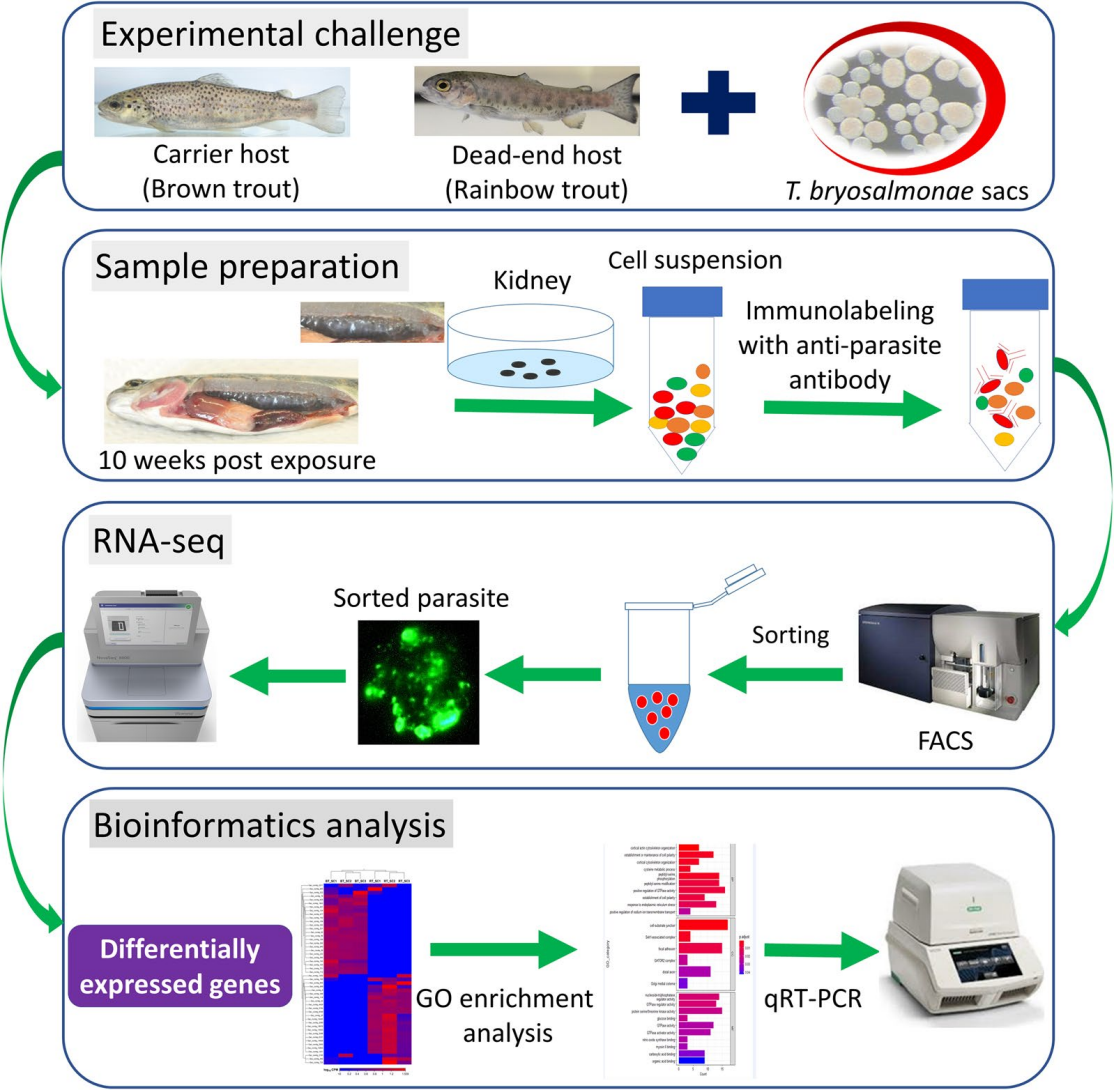
**Flowchart of the experimental setup used to sort** ***Tetracapsuloides bryosalmonae*** **from infected kidney of fish and RNA sequencing.** Brown trout and rainbow trout were experimentally challenged with laboratory cultured *T. bryosalmonae*. Ten weeks post-exposure kidney samples were collected and cell suspension was made from them. Cell suspension from kidney was stained with anti-*Tetracapsuloides bryosalmonae* antibody and anti-mouse antibody Alexa 488 and then subjected to sorting by FACS Aria III cell sorter. Samples were then used for RNA extraction and cDNA library preparation followed by sequencing. Subsequently, data was analyzed using bioinformatics approaches to identify differentially expressed genes of *T. bryosalmonae* between the two fish species.

The experimental exposure of fish was carried out with parasite spores released from infected bryozoans, according to Kumar et al. [17]. Briefly, prior to parasite exposure, the fish were kept in an aquarium with 100 L of water. To this, a spore suspension from 30 mature parasite sacs was added and mixed properly to aquaria. The water flow in the aquaria was stopped for 6 h with vigorous aeration and then re-started very slowly overnight. Following this, both brown trout and rainbow trout (*n* = 69 per fish species) were randomly divided into 3 groups as three replicates (*n* = 23 in each aquarium). Similarly, three replicates for each fish species (*n* = 69 per fish species and n = 23 for each replicate group) were maintained as uninfected controls without adding parasite spores. Fish (*n* = 9) were randomly sampled at 2-, 4-, 6-, 8-, 10-, 12-, and 17-weeks post-exposure (wpe) from both infected and uninfected control groups. The presence of parasite was confirmed in the kidneys using histology and immunohistochemistry. At 10 wpe, numerous intra-luminal sporogonic and pre-sporogonic stages (Figures 1A and 2A) in brown trout and interstitial pre-sporogenic stages of parasite in rainbow trout (Figures 1B and 2B) were observed. A description of experimental method for FACS and RNA-seq is presented in a flowchart (Figure 3).

### Sample preparation

During preliminary studies, the sample preparation protocol for the FACS analysis was optimized. Uninfected and infected kidney samples were used for the optimization of staining protocol. Different concentration of primary antibody, secondary antibody, incubation time, and temperature were tested. Additionally, during optimization a primary antibody alone control, secondary antibody alone control, unstained and stained (using both primary and secondary antibodies) uninfected control and infected kidney samples were tested before carrying out the following protocol. We used a combination of Dulbecco’s phosphate buffered saline (DPBS, Sigma) and 2% newborn calf serum (NCS) as blocking buffer. NCS was used to promote cell viability while also acting as a blocking agent. A portion of posterior kidney of sampled uninfected control and infected groups at 10 wpe were excised and placed in a Petri dish filled with pre-chilled DPBS. Each kidney sample was washed three time with cold DPBS to remove fat and blood clumps. Kidney sample was cut into small pieces (2–4 mm) with the scalpel and was washed 3 times with PBS containing 2% newborn calf serum (NCS) and kept at 4 °C until further processing. Each kidney tissue was disrupted manually with gentle pressure using cell strainer (70 μm) and a syringe barrel and the volume was made up to 8 mL with cold DPBS containing 2% NCS. The cell suspension was centrifuged at 2000 rpm for 10 min at 4 °C. The supernatant was discarded, and the pellet was resuspended in 1 mL of DPBS containing 2% NCS solution. The cells were counted with hemocytometer. Afterwards, cell suspension (4 × 10^6^ cells) was distributed to 1.5 mL Eppendorf tube and then centrifuged at 2000 rpm for 10 min at 4 °C. The supernatant was discarded, and the cell pellet was resuspended in 150 µL of the commercially available anti-*T. bryosalmonae* monoclonal antibody (1:10 diluted with cold DPBS containing 2% NCS) (Aquatic diagnostics ltd., Stirling, Scotland, UK) and incubated for 60 min at 4 °C. After incubation, cell suspension was washed two times with 800 µL of cold DPBS containing 2% NCS and centrifuged at 2000 rpm for 10 min at 4 °C. Subsequently, cell pellet was incubated with 100 µL of anti-mouse antibody Alexa 488 and incubated for 30 min and then washed two times with cold DPBS containing 2% NCS. Finally, cell pellet was resuspended in 400 µL of DPBS containing 2% NCS and then transferred in a FACS tube through tube filter and kept on ice for until use.

### Parasite cell sorting from infected kidney

A FACS Aria III cell sorter (Becton Dickinson, San Jose, USA) equipped with a 488 nm blue solid laser was used for analysis. Forward-scatter characteristics (FSC) resulted from the small-angle scatter, while side-scatter characteristics (SSC) were recorded as orthogonal scatter of the 488 nm laser. Fluorescence (eYFP/Venus) was detected by a 502-nm long-pass and a 530/30 nm band-pass filter set. The FACS software DIVA 7.0 was used for the recording. Thresholding on the FSC/SSC was applied for all measurements and removed for the sorting procedure. Initially, to enable gate settings uninfected kidney cell suspension were analysed. Cell sorting was achieved by pre-gating the cells twice. FSC and SSC characteristics were used to set the first gate to remove non-cell particles. The next gate was set around the FSC height and fluorescence area signal to select stained population of cells. From these pre-gating settings, the cells were sorted with the four-way purity mask and a threshold rate of 8000–25 000 events/s with 70 μm nozzle. Cells were sorted directly into 1.5 mL Eppendorf tubes containing cold lysis buffer with beta-mercaptoethanol (Qiagen, Germany) and the sorted cells were stored immediately at −80 °C. The parasite viability was examined visually under the fluorescence microscope before and after cell sorting.

### RNA extraction, library preparation and sequencing

Total RNA was extracted from FACS-sorted *T. bryosalmonae* from brown trout and rainbow trout using RNeasy UCP Micro Kit (Qiagen) along with an on-column DNase digestion step following the manufacturer’s protocol. RNA integrity was assessed on the 4200 TapeStation with the High Sensitivity RNA ScreenTape Kit (Agilent, Santa Clara, CA, USA). Only samples with RNA integrity numbers > 8.0 were used for further analysis.

Library preparation was done with 60 ng total RNA input using the Poly(A) RNA Selection Kit V1.5 and the CORALL mRNA-Seq Library Prep Kit (Lexogen, Vienna, Austria) according to the manufacturers protocol. Six cDNA libraries (3 each for sorted parasite samples from brown trout and rainbow trout) were prepared. Library quality control was done with the High Sensitivity D1000 ScreenTape Kit on the 4200 TapeStation (Agilent). Libraries were sequenced on a NovaSeq 6000 system (Illumina, San Diego, CA, USA) implementing 150-bp paired-end reads. Sequencing was done by the NGS unit of the Vienna Biocenter Core Facilities (VBCF, Vienna, Austria).

### Analysis of RNA-seq data

Data were analyzed with CLC Genomics Workbench software v22 (Qiagen, Aarhus, Denmark). The raw reads were subjected to quality filtering: low quality bases (Phred score ≤ 30), reads shorter than 50 nucleotides, adapter and unique molecular identifier sequences from library prep were removed. To remove host sequences, the filtered reads were mapped to either brown trout (NCBI accession: GCA_901001165.2) or rainbow trout genome (GCA_013265735.3) with the CLC mapping tool using the default mapping parameters (mismatch cost = 2, insertion cost = 3, deletion cost = 3, length fraction = 0.8, similarity fraction = 0.8). Three publicly available transcriptome assemblies from *T. bryosalmonae* [18–20] were combined into one file and redundant sequences were removed using the clustering software cd-hit-est with a sequence identity threshold of 0.95 [21]. The remaining unmapped reads were mapped to the available myxozoan genomes and the combined, non-redundant *T. bryosalmonae* transcriptome assembly. The details of datasets used for mapping are provided in Table 1.

**Table 1:** **Summary of mapping data of FACS sorted** ***Tetracapsuloides bryosalmonae***

Samples	Number of raw reads	Number of quality-filtered reads	Number of reads mapping to brown trout or rainbow trout	Remaining: number of unmapped reads	Mapping of unmapped reads: number of mapping reads to different references							
					*E. leei* mitochondrial genome (5 genes) GCA_001455295.2	* K. iwatai* mitochondrial genome (10 genes) GCA_001407235.2	*M. squamalis* (5710 genes) GCA_010108815.1	* H. salminicola* (8187 genes) GCA_009887335.1	* S. zaharoni* (number annotated genes) GCA_001455285.1		*T. kitauei* (15 020 genes) GCA_000827895.1	Combined *T. bryosalmonae* transcriptome (31 464 contigs, Ahmad et al. [20]; Faber et al. [19]; Kumar et al. [18]
BT_SC1	71 360 140	68 363 654	43 666 912	24 696 742	2 308 164	3 837 812	2 121 654	2 121 654	2 383 909		3 297 048	13 551 795
BT_SC2	76 160 696	73 576 346	44 245 459	29 330 887	2 936 500	4 862 586	2 656 946	2 656 946	3 017 066		4 109 527	16 276 440
BT_SC3	80 297 000	76 804 534	48 592 048	28 212 486	2 495 135	4 166 910	2 332 754	2 332 754	2 576 958		3 586 536	15 870 805
RT_SC1	59 057 686	56 787 460	37 180 648	19 606 812	1 991 590	3 036 937	1 659 712	1 659 712	1 859 904		2 641 455	8 123 191
RT_SC2	87 380 018	83 725 584	51 212 973	32 512 611	3 551 579	5 591 392	2 989 800	2 989 800	3 505 367		4 802 022	14 838 808
RT_SC3	72 073 626	68 923 366	48 272 618	20 650 748	1 801 703	2 812 857	1 467 204	1 467 204	1 770 479		2 411 495	7 777 966

The filtered clean reads of sorted *T. bryosalmonae* obtained from infected kidney of brown trout and rainbow trout were mapped to different genome and transcriptome references (fish hosts and myxozoan parasites). The remaining unmapped reads were mapped against the genomes of different myxozoan parasites and the combined transcriptome of *T. bryosalmonae* from brown trout, rainbow trout and bryozoan hosts. RNA-seq analysis was performed using CLC Genomics Workbench software version 22. *T. bryosalmonae* samples from brown trout: BT_SC1, BT_SC2, BT_SC3; *T. bryosalmonae* samples from rainbow trout: RT_SC1, RT_SC2, RT_SC3.

Venn diagram was constructed using the online tool InteractiVenn [22] to exhibit the number of shared and unique transcripts of *T. bryosalmonae* in brown trout and rainbow trout. The parasite transcripts (average RPKM > 1.0) from brown trout and rainbow trout were used to construct the Venn diagram. For differential expression, brown trout samples (*n* = 3) were compared to rainbow trout samples (n = 3). Transcripts showing absolute fold changes > 2.0 and FDR-corrected *p*-values < 0.01 were considered as differentially expressed. The calculation of p-values implements the “exact negative binomial test” proposed by Robinson and Smyth [23]. Heatmaps of differentially expressed transcripts were generated with the single linkage method based on Euclidean distance matrix. Heatmap and volcano plot were generated with the SRplot online tool [accessed on 21 March 2023]. GO categories (biological process, molecular function, and cellular components) enriched in DETs were identified using the clusterProfiler package enrichGO in R (version 4.2.1) [24]. For each comparison, upregulated and downregulated transcript sets were input separately into enrichGO. A *p-*value cut-off of 0.05 was used. All quality control steps followed in this study right from sample preparation to RNA-seq data analysis are provided in Additional file 1.

### Validation of differentially expressed transcripts by quantitative real time PCR

To test the validity of RNA-seq results, we performed a quantitative analysis of six randomly selected differentially expressed transcripts (Additional file 2) by quantitative real-time PCR (qRT-PCR) on both RNA extracted from sorted cells as well as from infected kidney samples. The analysed transcripts included ATP-binding cassette sub-family G member 4-like, gag-pol fusion protein, leukocyte surface antigen CD53-like, predicted protein, CD63 antigen, NHP2-like protein 1. Transcript-specific primers were designed using the online tool NCBI Primer-BLAST. Gradient PCR was used to evaluate optimum annealing temperature of designed primers, and serial dilutions were used to test each transcript’s primer efficiency. To further ensure their specificity and sensitivity, each primer set’s distinctive amplicon was sequenced and subjected to a BLAST analysis. Sixty ng of total RNA from each sorted sample and 1 µg of RNA from infected kidney samples was used to synthesize cDNA using iScript cDNA Synthesis Kit (Bio-Rad, Hercules, USA). The cDNA samples (*n* = 3) of *T. bryosalmonae* from brown trout and rainbow trout were subjected to qRT-PCR with two technical replicates using the optimized primers. qRT-PCR was performed in a final volume of 10 µL, which contained 3 µL parasite sorted cells and infected kidney diluted cDNA samples, 0.5 µM of each primer, 1× SsoAdvanced™ Universal SYBR Green Supermix (Bio-Rad) and DEPC-treated sterile distilled water. After 5 min of cDNA denaturation at 95 °C, 37 cycles were performed at 95 °C for 30 s, annealing at 58–60 °C for 30 s and 72 °C for 30 s in a CFX96 Touch Real-Time PCR detection system (Bio-Rad). At the end of all gene expression cycling protocols, melting curve analysis was performed to validate amplification specificity under the following conditions: 58–60 °C for 30 s to 95 °C with an increment of 0.5 °C for 10 s. CWF19-like protein 2 and RPL-18 [19] were used as reference genes to normalize the test samples. The 2^−ΔΔCt^ method was calculated to determine the relative gene expression of *T. bryosalmonae* from the brown trout group relative to the rainbow trout group. The statistical difference between groups was determined using unpaired Student’s *t*-test. Linear regression analysis was performed on corresponding log_2_ fold change values of RNA-seq and qRT-PCR to evaluate the relationship between them. For all statistical tests, *P*-value of < 0.05 was regarded as significant.

## Results

### Isolation of *T. bryosalmonae* from infected kidney

**Figure 4:**
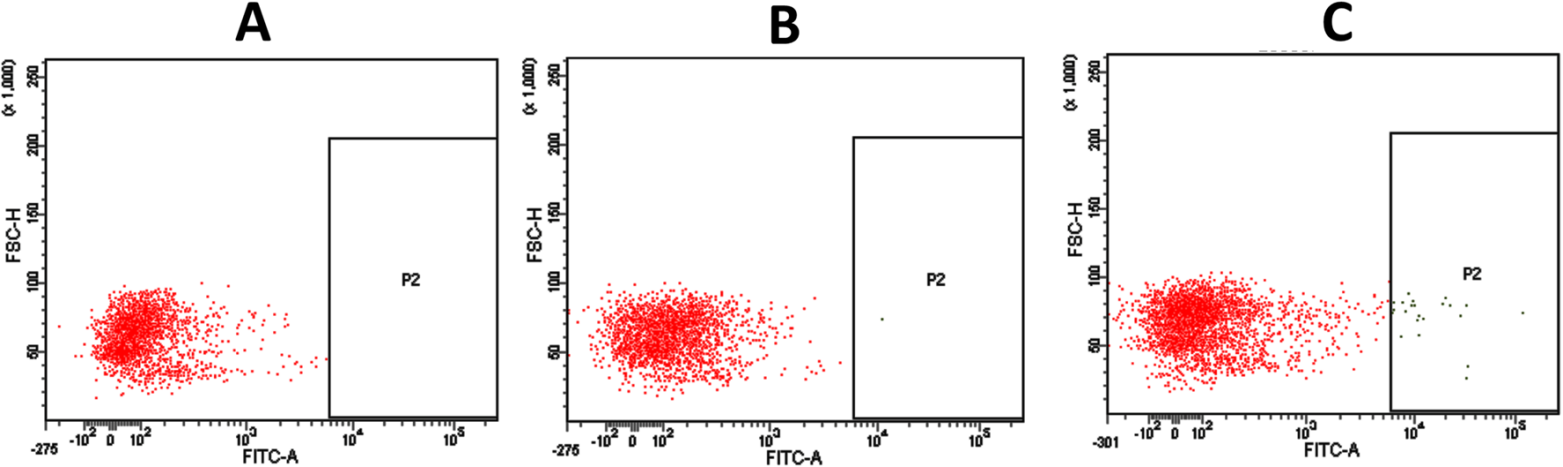
**Isolation of**  ***Tetracapsuloides bryosalmonae*** **by FACS Aria III cell sorter.** Figures represent a two-dimensional scatterplot which corresponds to Fluorescein isothiocyanate area (FITC-A) versus forward scatter height (FSC-H). **A** Unstained infected kidney cells as control; **B** stained infected kidney cells with anti-mouse antibody Alexa 488 as a secondary antibody alone control; **C** stained infected kidney cells with anti-*T. bryosalmonae* monoclonal antibody and anti-mouse antibody Alexa 488.

**Figure 5:**
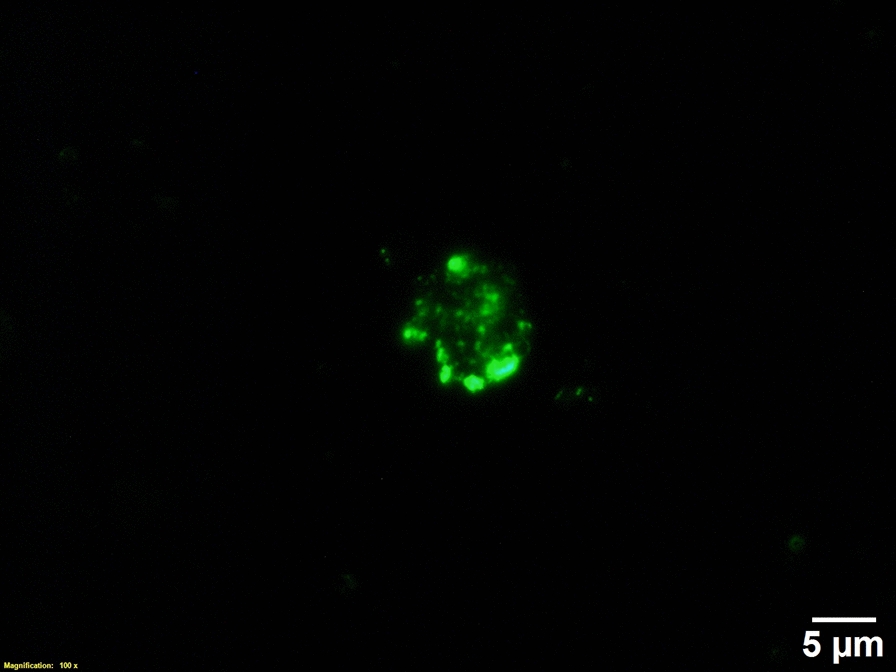
**The sorted cell of**  ***Tetracapsuloides bryosalmonae.*** Cell suspension from infected kidney was stained with anti-*T. bryosalmonae* monoclonal antibody and anti-mouse antibody Alexa 488 and then subjected to sorting by FACS Aria III cell sorter.

To isolate parasites from the infected kidney cell suspension of brown trout and rainbow trout, the *T. bryosalmonae* populations were characterized using the representative two-dimensional scatterplots. The scatterplots in Figures 4A–C represent forward scatter height (FSC-H) as a function of the fluorescein isothiocyanate area (FITC-A). Unstained infected kidney cells and secondary antibody alone stained infected cells respectively, allowed to detect and eliminate autofluorescence of different cell types (Figure 4A) and non-specific binding (Figure 4B). Figure 4C shows the distribution of parasites within the rectangular gate from stained infected cells. Each dot inside this gate is considered as one event defined as a parasite cell. For infected kidney samples from each fish host, parasites were always sorted from the rectangular gate. The parasite cell after sorting is demonstrated in Figure 5.

### RNA-seq analysis

Sequencing of the six libraries yielded 446.32 million raw reads. The number of raw reads from each library ranged from 59.05 to 87.38 million (Table 1). After quality filtering, 56.78–83.72 million reads were retained. Following mapping to brown trout and rainbow trout genomes, 82.24 (37.59%) and 72.77 million reads (34.74%) remained, respectively. After mapping to myxozoan genomes and the *T. bryosalmonae* transcriptome the highest mapping percentage was obtained with the combined *T. bryosalmonae* transcriptome assembly. Therefore, we used the combined *T. bryosalmonae* transcriptome assembly as a reference for mapping of RNA-seq data. 15.2 million and 10.2 million average parasite reads mapped from brown trout and rainbow trout respectively (Table 1). These reads were assembled into 4039 and 8037 contigs of *T. bryosalmonae* (transcripts with average RPKM > 1.0) from rainbow trout and brown trout, respectively. The expression values (read counts and RPKM) for all parasite transcripts is provided in Additional file 3.

**Figure 6:**
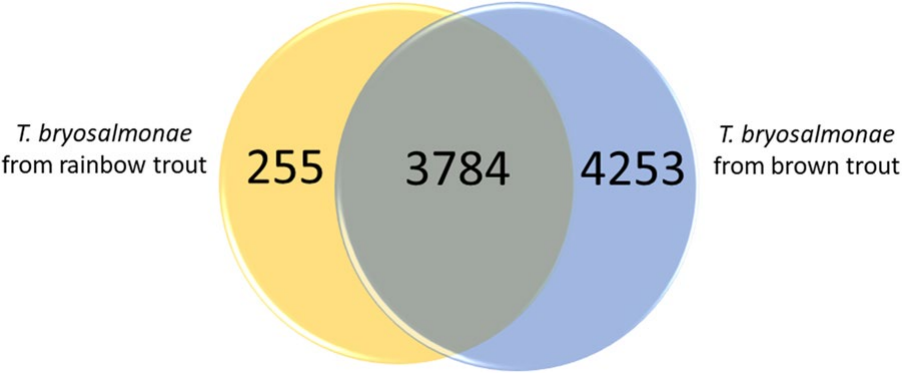
**Venn diagram of total identified transcripts of** ***Tetracapsuloides bryosalmonae.***. Venn diagram representing number of unique and shared genes in *T. bryosalmonae*. The number of common genes of *T. bryosalmonae* between the brown trout and rainbow trout was 3784, whereas 255 and 4253 genes were found to be unique to rainbow trout and brown trout, respectively.

The Venn diagram showed 3784 shared parasite contigs between the two fish hosts, whereas 255 and 4253 transcripts were found to be unique in rainbow trout and brown trout, respectively (Figure 6).

### Differential transcript analysis

**Figure 7:**
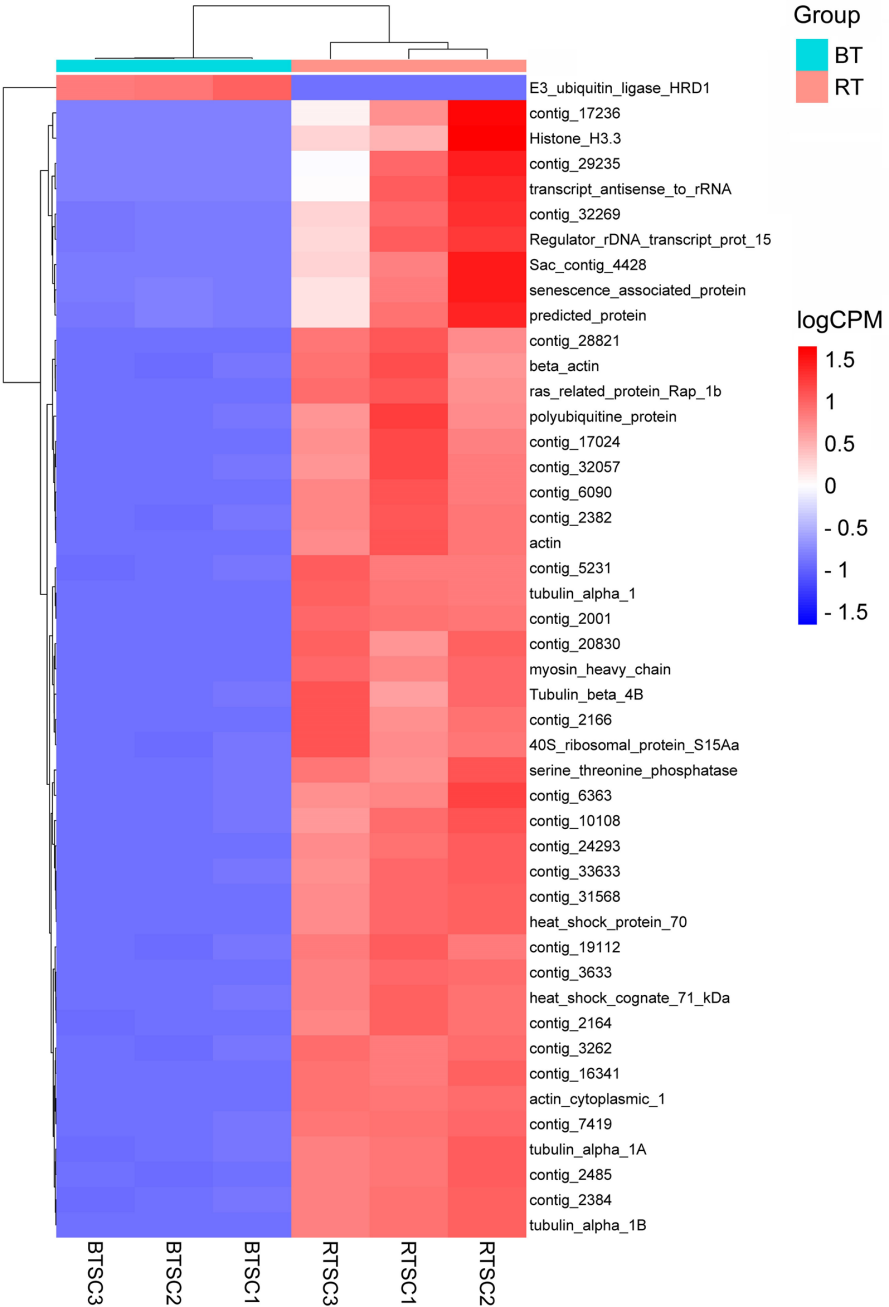
**Heatmap visualization and hierarchical clustering of top 50 differentially expressed transcripts.** The heatmap shows 50 differentially expressed transcripts of *Tetracapsuloides bryosalmonae* between brown trout and rainbow trout selected based on FDR adjusted *P*-value. Hierarchical clustering was performed using the single linkage method based on Euclidean distance matrix. The columns represent *T. bryosalmonae* samples and rows represent selected genes.

**Figure 8:**
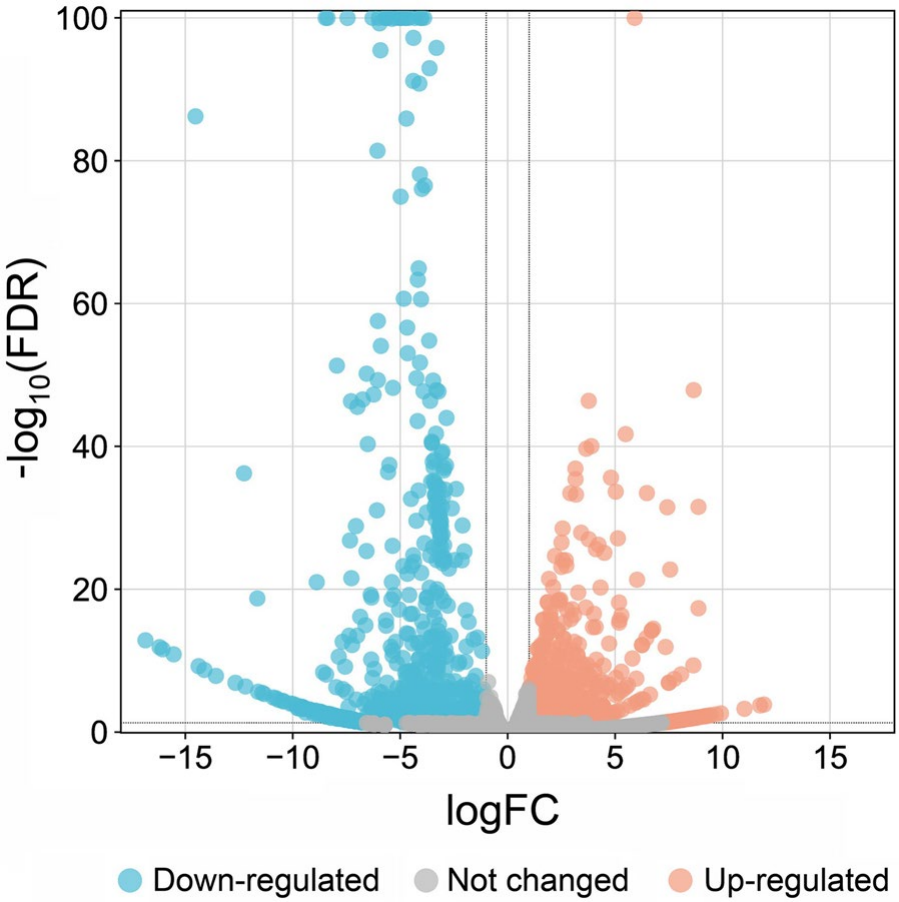
**Volcano plot of**  ***Tetracapsuloides bryosalmonae*** **transcripts in brown trout compared to rainbow trout.** The x-axis shows log_2_ transformed fold change, and the y-axis shows log_10_ transformed adjusted significance. Each dot represents an individual gene that is significantly downregulated (blue), significantly upregulated (red) or unchanged (grey) in brown trout compared to rainbow trout. The dashed horizontal line represents the 0.01 cut-off for adjusted *P*-value, and the dashed vertical lines represent the fold change cut-off of two.

Comparative transcript expression analysis between *T. bryosalmonae* from brown trout and rainbow trout revealed 1120 DETs of *T. bryosalmonae* (fold change > 2 or < −2, FDR *p*-value < 0.01). This accounts for 3.6% of the total contigs available in the reference *T. bryosalmonae* transcriptome. DETs were visualized in a heatmap and a volcano plots (Figures 7 and 8). Out of 1120 DETs, 548 transcripts (48.93%) were upregulated, and 572 transcripts (51.07%) were downregulated (Additional file 4).

### Gene ontology

**Figure 9:**
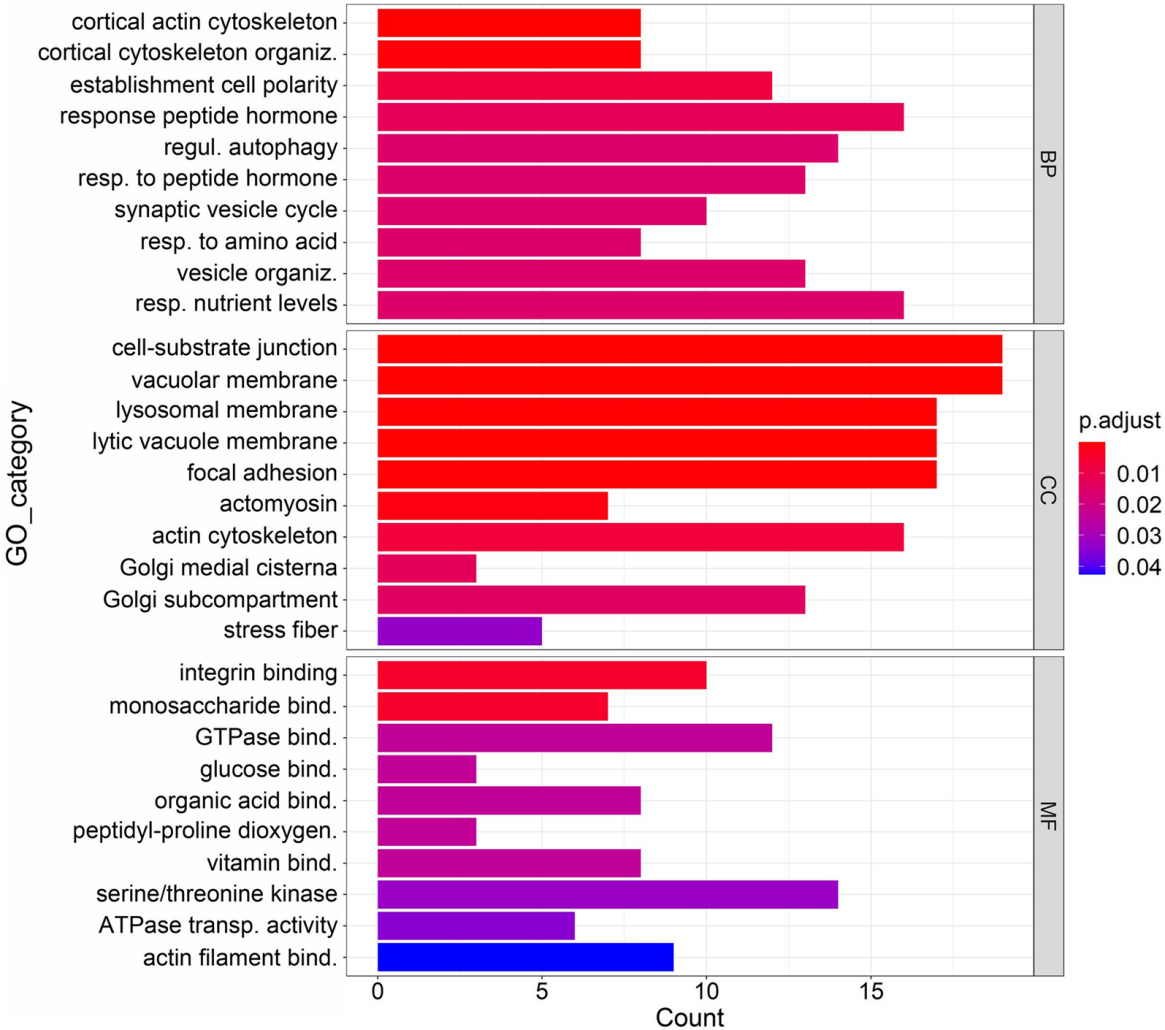
**Functional enrichment analysis of upregulated DETs shown by bar plot.** Three sub ontologies of GO enrichment analysis: the biological process (BP), cellular component (CC) and molecular function (MF). GO terms are represented on y-axis and number of genes (count) in each GO term are represented on x-axis.

Out of the identified 1120 DETs, 371 transcripts (33.12%) were assigned to gene ontology terms. The important biological processes with which upregulated transcripts were associated included cortical actin cytoskeleton organization, establishment or maintenance of cell polarity, and peptidyl-serine phosphorylation and modification. The upregulated transcripts were part of cellular components such as cell substrate junction, focal adhesion, and golgi medial cisterna. The upregulated transcripts were involved in molecular functions such as integrin binding, glucose binding, protein serine kinase activity, GTPase binding, and peptidyl-proline dioxygenase activity (Figure 9).

**Figure 10:**
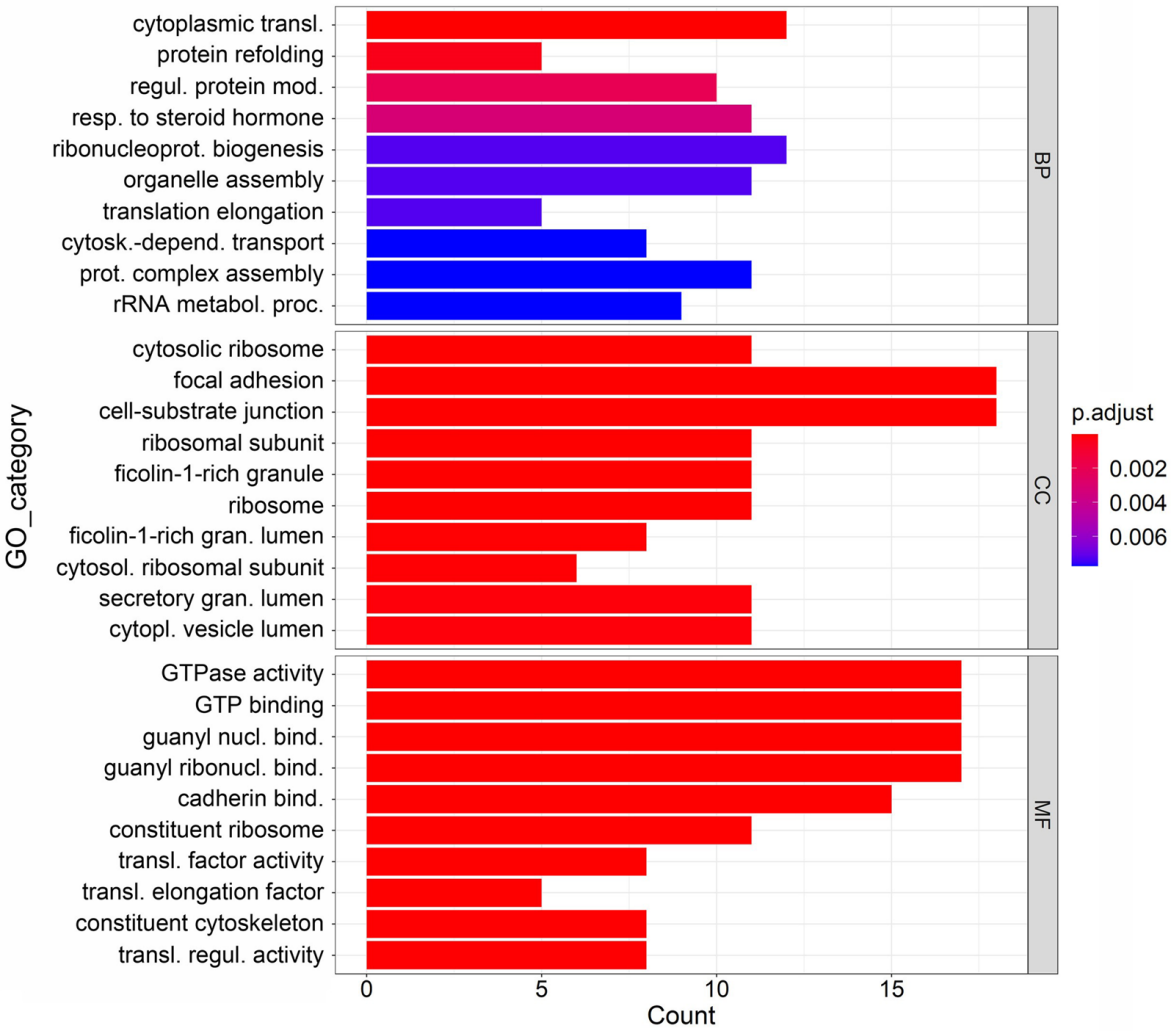
**Functional enrichment analysis of downregulated DETs shown by bar plot.** Three sub ontologies of GO enrichment analysis: the biological process (BP), cellular component (CC) and molecular function (MF). GO terms are represented on y-axis and number of genes (count) in each GO term are represented on x-axis.

The downregulated transcripts were associated with the biological processes such as cytoplasmic translation, ribonucleoprotein complex biogenesis, subunit organisation and assembly, translational elongation, regulation of translation, non-membrane bounded organelle assembly, and regulation of protein cellular catabolic process. The cellular component analysis of these transcripts indicated that they belonged to the following GO categories: focal adhesion, cell-substrate junction, ribosomal subunit, ficolin-1-rich granule, secretory granule lumen and cytosolic ribosome. In addition, the downregulated transcripts have molecular functions such as GTPase activity, GTP binding, cadherin binding, guanyl nucleotide binding, structural constituent of ribosomes and cytoskeleton and translation regulator activity (Figure 10).

### Validation of selected transcripts

**Figure 11:**
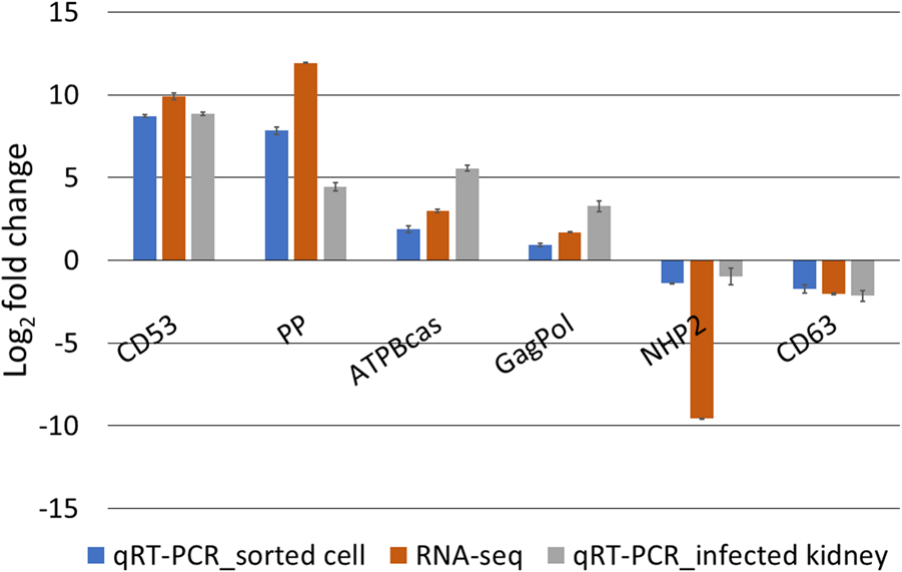
**Validation of RNA-seq analysis by qRT-PCR on selected genes.** The gene expression values are represented as relative log_2_-fold change (mean ± SEM) of *Tetracapsuloides bryosalmonae*-brown trout group compared to the rainbow trout group (*n* = 3). *CD53:* leukocyte surface antigen CD53-like, *PP* predicted protein: *ATPBcas:* ATP-binding cassette sub-family G member 4-like, *GagPol:* gag-pol fusion protein, *NHP2:* NHP2-like protein 1, and *CD63:* CD63 antigen. *qRT-PCR:* Quantitative Real time Polymerase chain reaction, *RNA-seq:* RNA sequencing.

Expression profiles of the randomly selected transcripts by qRT-PCR were consistent with the patterns of expression revealed by the RNA-seq (Figure 11). The parasite transcripts that were upregulated and downregulated by RNA-seq and qRT-PCR of sorted cells showed similar expression pattern in the fish kidney tissue, thereby confirming the reliability of our approach.

## Discussion

The behavior and outcome of *T. bryosalmonae* within hosts can be profoundly influenced by a myriad of host-associated factors, highlighting their indispensable role in shaping the dynamics and ultimate outcomes of the biological system. However, the differential behavior of parasite between both the hosts can also be a response of the parasite itself and the focus of this study was to look into such parasite associated factors. We conducted this study to identify differentially expressed transcripts of *T. bryosalmonae* between brown trout (carrier host) and rainbow trout (dead-end host). We used FACS to obtain pure parasites in large numbers from infected kidney of both the fish species. To our knowledge, this is the first study reporting the isolation of *T. bryosalmonae* from fish host by FACS. The commercially available monoclonal antibody against *T. bryosalmonae* that was used for staining cells has been found to identify both pre-sporogonic stages and sporogonic stages of the parasite [25]. Subsequent to FACS isolation, sorted parasite cells were subjected to RNA-seq. In recent years, RNA-seq is being widely used for generating huge amount of transcriptome data for many myxozoan parasites, the analysis of which is leading to impactful information [14]. Concerning *T. bryosalmonae*, some of the previous studies utilized dual RNA-seq to study its transcriptome from infected kidney of brown trout [20], and the overlap transcriptome between infected kidney of rainbow trout and bryozoan hosts [19]. However, the comparative transcriptome of this parasite between infected kidney of rainbow trout and brown trout has not been attempted earlier. This is remarkable considering the disparity in the fate of the parasite in these hosts; successful completion of life cycle in brown trout whereas clearance or degradation in rainbow trout [26]. Therefore, in our work, we applied RNA-seq to analyze the comparative transcriptome of FACS purified *T. bryosalmonae* from brown trout and rainbow trout. From our approach, we obtained more than twice host-filtered reads as reported by Faber et al. [19] through dual RNA-seq of infected kidney of rainbow trout. Similarly, we obtained greater than 10 times host-filtered reads as reported by Ahmad et al. [20] from infected kidney of brown trout. This is despite the fact that the total number of reads obtained by Faber et al. [19] and Ahmad et al. [20] was 1.6 and 2 times higher than what we found. This suggests towards the enrichment of parasite reads in our study by using FACS. Nevertheless, we too had 62–65% host reads. However, this might be related to the close association of *T. bryosalmonae* with host macrophages [7], also shown in the histology figure from our study (Figure 1B). Additionally, a cursory glance at the host mapped reads also showed that many genes are macrophage associated (such as interleukin, nitric oxide synthase, Zinc Finger CCCH-Type Containing 12 A, Nuclear Factor Kappa B Subunit 2, Retinal Dehydrogenase 2), which could be a possible explanation for the presence of host reads. Our study revealed many differentially expressed transcripts, likely to be involved in the fundamental processes required for its survival in the fish host. Understanding these differences in transcript expression of *T. bryosalmonae* in brown trout, and rainbow trout can be instrumental in future development of control measures.

In comparison to the dead-end host, *T. bryosalmonae* seemingly reflect an adaptive plastic strategy in the carrier host. Parasites are detectable in blood initially during the pre-clinical [9, 27], and later in the post-clinical phase of infection in brown trout [28]. Additionally, probably the parasites are maintained throughout their lives in this host [28, 29]. On the contrary, in rainbow trout the parasites are suggested to be cleared in the fish surviving the infection [30]. From the host perspective, B cell and Th1-like cytokines mediated immune responses probably confer greater tolerance and lower resistance to brown trout in comparison to rainbow trout [26]. However, information on the response of the parasite at the molecular level in both fish hosts are obscure. Nevertheless, there is widespread recognition that transcriptional modulation is critical for the survival of parasites in order to adapt to host immune responses and physiological changes [31]. Accordingly, it is plausible that the parasite is able to adapt to the host immune response in brown trout by varying its gene expression, whereas it cannot do so adequately or appropriately in rainbow trout [26]. In the present work, we experimentally infected both brown trout and rainbow trout with the same *T. bryosalmonae* lineage, indicating that the parasite had to adapt its gene expression levels in each of the fish to overcome immune defences and differences in the physiological conditions in the host. Changes in the *T. bryosalmonae* transcriptome can hence, be directly linked to the different host environments and immune responses, which is supported by the biological function of differentially expressed transcripts. Table 2 summarises some of the important physiological functions and the differentially expressed parasite transcripts possibly associated with them. Here, we attempt to highlight and discuss some of the enriched biological processes of DETs, which might be crucial for parasite development in the fish host and require closer examination.

**Table 2:** **Some major physiological functions of parasites and associated differentially expressed genes found in this study**

Function	Gene	Log_2_ fold change (*p* value < 0.001) *T. bryosalmonae* (brown trout vs. rainbow trout)
Adherens junctions linking proteins	Catenin beta-like	−3.879
Actin polymerization	ras-like GTP-binding protein Rho1 isoform X2	−3.985
	ras-related GTP-binding protein A	2.796
	ras-related protein Rac1	−14.379
	T-cell activation Rho GTPase-activating protein	−1.711
	rho GTPase-activating protein 5 isoform X1	1.361
	rho GTPase-activating protein 39-like	1.948
	rho-associated protein kinase 1 isoform X1	1.980
	Afadin isoform X5	5.159
Adhesion molecules	Putative alpha P integrin isoform X1	−3.688
	Neural cell adhesion molecule 2	5.028
Tight junction proteins	Tetraspanin-9	1.769
	Tetraspanin-4	2.245
Extracellular matrix	fibronectin type-III domain-containing protein 3 A isoform X1	1.295
	collagen alpha-1 (XII) chain	3.639
	matrix metalloproteinase-25-like	3.271
Integrin-cytoskeleton links	talin-1 isoform X1	1.55
	alpha-actinin-1 isoform X3	2.113
	alpha-actinin, sarcomeric-like isoform X2	−5.290
	AChain A, X-ray structure of the human alpha-actinin isoform 3	−3.039
Nucleotide transporter	adenosine 3’-phospho 5’-phosphosulfate transporter 1	4.3565
Amino acid transporters	ATP-binding cassette sub-family G member 4-like	1.801
	ATP-binding cassette sub-family A member 12	1.537
	high affinity cationic amino acid transporter 1-like	3.088
	cationic amino acid transporter 2-like	3.310
Ion transporters (channel/Pore type transporters)	solute carrier organic anion transporter family member 4A1 isoform X2	−1.594
	vesicular glutamate transporter 2-like	2.149
	voltage-dependent calcium channel type A subunit alpha-1-like	4.013
	voltage-dependent N-type calcium channel subunit alpha-1B-like isoform X1	5.495
Ion transporters (ATP driven Na + dependent or cotransporters	Kanadaptin	1.478
	putative glycerol-3-phosphate transporter 5	1.407
Cell signalling	EGF-like domain-containing protein	6.451
	CREB-binding protein	−5.874

The table describes some of the key physiological functions of differentially expressed transcripts identified in this study. The log_2_ fold change of the parasite transcripts between brown trout and rainbow trout at a *p* value < 0.001 are presented.

### Evidence of different gene regulatory mechanisms in *T. bryosalmonae*

Like all organisms, the survival of parasites depends on stringent spatiotemporal regulation of gene expression finally leading to the synthesis of diverse proteins that are the principal functional units. The translation of mRNAs into proteins is dependent on many other RNAs, ribosomal proteins and protein-RNA complexes. In our study, the GO analysis of downregulated DETs of *T. bryosalmonae* (from brown trout compared to rainbow trout) revealed that the top ten GO terms were mainly processes associated with translation and proteins. The cells can respond to environmental cues much faster through translational control rather than transcriptional regulation [32]. This is because the easiest strategy to regulate the protein function over time is to inhibit or promote its synthesis. This might be significant for *T. bryosalmonae*, which has a complex life cycle undergoing different developmental stage transitions in its fish hosts during which gene expression regulation might occur essentially at the post-transcriptional level.

The occurrence of post-transcriptional regulation is supported by the presence of many of the genes among the DETs. For instance, argonaute genes, the component of RNA interference (RNAi) machinery [33], as well as protein phosphatase 1 catalytic subunit that play key roles in post-transcriptional gene regulation [34]. Among the different mechanisms, RNA degradation is an important method of post-transcriptional regulation [35]. Exosome complex (EXOSC) genes are important mediators of RNA degradation pathway that encode a multi-protein intracellular complex [36]. We found downregulation of EXOSC7 and its association with the GO category of regulation of translation. The mature spores of another myxozoan parasite, *C. shasta* were found to contain EXOS1 and EXOS2 [37]. These findings hint at important roles of EXOSC genes in the maturation of myxozoan spores. Beside these genes, the presence of CPEB [38], RACK1 [39], and CNOT1 [40], probably emphasise major roles of post-transcriptional as well as post-translational regulation during the parasite development in the fish host.

### Role of ribonucleoprotein complex (RNPs) in parasite gene regulation

Interestingly, our data revealed that many downregulated DETs were associated with the ribonucleoprotein complex biogenesis and ribonucleoprotein complex subunit organization categories. Ribonucleoprotein complex (RNPs) play important role in determining the fate of RNA in eukaryotes. RNPs consist of mRNA bound to RNA-binding proteins (RBPs). In addition to providing protection to the RNA molecule, RNPs are involved in several processes during its transport from the site of transcription to the site of translation in the cytoplasm. Ribosomal proteins are involved in regulation of gene-specific transcription and translation processes [41]. Additionally, alternative ribosome variants help eukaryotic cells to adapt to changing conditions [42]. Currently information on gene regulatory mechanisms in myxozoan parasites is scarce and needs to be investigated.

### Downregulated biological process of cytoplasmic translation

The biological process of cytoplasmic translation was the top process related to the downregulated DETs. A possible explanation for this could be that downregulation of genes related to translation in *T. bryosalmonae* from brown trout might be a probable parasite strategy similar to other parasites such as *Plasmodium.* These parasites employ translational delay wherein protein expression is actively suspended for expressed mRNA transcripts to quickly adapt to new environments and undergo developmental switching facilitating their survival. Storing transcripts required for such adaptations allows for rapid changes in gene expression by bypassing the time needed for transcription [43]. Additionally, many coccidian protozoan organisms are known to regulate the transcription of ribosome biosynthesis in order to adapt to changes that accompany stage transitions during their developmental life cycles [44, 45]. Previous studies have reported the presence of *T. bryosalmonae* sporogonic stages in the kidney lumen of brown trout but not in rainbow trout [25]. These findings are suggestive of employment of translational delay by *T. bryosalmonae* as an immune evasion mechanism and a strategy for their development from extrasporogonic to sporogonic stages in brown trout when compared to rainbow trout. Nevertheless, functional studies would be required to validate this notion.

### Expression of transcripts related to formation of processing bodies (P bodies)

An interesting enriched biological process was non-membrane-bounded organelle assembly, which refers to the aggregation and arrangement of a variety of biomolecules to form a non-membrane-bounded organelle. These cellular compartments tend to assemble and disassemble rapidly. Some important organelles belonging to this category include nucleoli [46], P-granules [46], Cajal bodies [47], stress granules [48] and signalling complexes on the cytosolic face of membranes [49]. Each of these organelles have a unique molecular composition despite sharing some similarities. Processing bodies (P bodies) are primarily composed of mRNAs in complex with proteins associated with translation repression and 5′-to-3′ mRNA decay (e.g., deadenylation complex Ccr4-Not, decapping activators DDX6). Recently, myosin (MYO1C, MYO1D, MYO6 and MYH10) have been identified as P- body proteins [50]. In our study, the presence of genes (e.g., CNOT1, MEF2A and MYH6), linked to the biological process of non-membrane-bounded organelle assembly might indicate the presence of P bodies. Different functions have been proposed for P bodies such as storage, decay and release of mRNA upon stress removal [51]. The formation of these organelles increases in response to diverse stress stimuli and disappear during mitosis. In view of the proposition of coevolution of *T. bryosalmonae* with brown trout and not with rainbow trout, the downregulation of DETs related to the tentative P bodies is expected in the former host. Nevertheless, there is no information on the formation of RNP granules in *T. bryosalmonae*, as well as in any of the myxozoans in general.

### *T. bryosalmonae* heat shock proteins (HSPs)

In the present study, the GO term protein refolding was one of the significant biological processes in downregulated DETs. Genes linked with this process included HSP70, HSP90 and HSP60 family members. Parasites express HSPs in response to diverse stimuli such as heat and oxidative stress that confer them resistance to these harsh conditions and are therefore critical to their survival [52]. Additionally, HSPs play important roles in several other processes such as protein homeostasis. They are known to bind to non-native forms of proteins to facilitate their folding to native conformations [53]. Both brown trout and rainbow trout are known to mount robust immune responses against *T. bryosalmonae* [26]. In turn, *T. bryosalmonae* need to overcome these challenges, which might be adversely affecting many of their physiological processes including protein structure and function. Thus, the upregulation of these parasite HSPs might be suggestive that they are the main strategy used by *T. bryosalmonae* for maintaining protein homeostasis in rainbow trout, whereas some other strategy might be employed solely or in combination with HSPs in case of brown trout. Additionally, HSPs in several parasites such as *T. cruzi* [54] and Schistosomes [55] have been found to be immunogenic in their hosts. In our recent study, we found the HSP70 and HSP90 of *T. bryosalmonae* to be immunogenic using in vivo induced antigen technology [56].

### Cytoskeleton organisation and cell polarity crucial for parasite development

GO analysis of upregulated differentially expressed transcripts of *T. bryosalmonae* (brown trout vs. rainbow trout) showed significant enrichment in the biological processes of cytoskeleton organisation and cell polarity. The actin cytoskeleton is a dynamic network playing important roles in many cellular mechanisms such as division, motility and shape maintenance in addition to generating mechanical forces within the cell [57]. In our study, the genes associated to the cytoskeleton organisation included talin 1 (TLN1), and erythrocyte membrane protein band 4.1 like protein 5 (EPB41L5). TLN1 is responsible for connecting integrins to actin and the regulation of integrin adhesion complexes [58]. Reportedly, this gene is involved in the motility of highly virulent *Ceratomyxa shasta* strains but not in avirulent strains [59]. Protein 4.1 [encoded by erythrocyte membrane protein band 41 (EPB41) gene] is a membrane-cytoskeleton protein cross-linker and adaptor that connects cytoplasmic spectrin-actin filament complexes and a wide variety of transmembrane proteins [60]. The erythrocyte membrane protein of *Plasmodium falciparum* is shown to promote adherence of infected host erythrocytes to microvascular epithelium [61] in a process called as sequestration which allows the parasite to escape the host immune response [62]. Besides, *P. falciparum* EMP1 has been demonstrated to adhere to brain, intestinal and kidney endothelial cells [63]. Earlier studies have reported the attachment of *T. bryosalmonae* to the vascular endothelium of host [64, 65]. Though unexplored in *T. bryosalmonae* immune evasion does occur as evident from persistence of the parasites in brown trout. Together, these findings suggest that EMPs might be an important player in the attachment of parasites to vascular endothelium and also to kidney cells, thereby in some way aiding in their evasion from host defenses. Therefore, the role of *T. bryosalmonae* EMPs needs to be further explored.

Cell polarity involves polarized organization of cell membrane-associated proteins and the asymmetric organization of organelles and cytoskeleton [66]. It governs diverse cellular processes as differentiation, localized membrane growth, directional cell migration, and vectorial transport of molecules across cell layers [67]. Under this GO term, we found many members of serine-threonine kinase family. Additionally, integrin linked kinase (ILK) was also associated to this process, which is known to play an important role in collective cell migration and establishment of cell polarity [68].

Extrasporogonic stages of *T. bryosalmonae* present in kidney interstitium migrate to kidney lumen by amoeboid movement for further development to form sporogonic stages [7]. While extrasporogonic stages of *T. bryosalmonae* have been reported in both brown trout and rainbow trout, sporogonic stages in renal lumen are reported only in the carrier host (Figure 1A). In myxozoan parasites, many studies have pointed towards the fundamental importance of motility during invasion, en route target tissue and during development in their hosts [69, 70]. Besides, the involvement of actin cytoskeleton in motility of this group of parasites is also demonstrated [59, 71, 72]. Consistent with these findings, we postulate that higher expression of *T. bryosalmonae* genes related to cytoskeleton organization and cell polarity in carriers as compared to dead-end hosts may be playing an important role in the development and differentiation of the extrasporogonic stages of the parasite to sporogonic stages in brown trout.

### Post-translational modifications (PTMs) of serine phosphorylation

Two interesting processes containing upregulated DETs were peptidyl-serine phosphorylation and peptidyl-serine modification. In eukaryotes, PTMs govern various essential functions including cell signaling, protein trafficking, epigenetic control of gene expression, cell-cell interactions, and cell proliferation and differentiation [73]. One of the most studied and important PTMs is phosphorylation [74]. Phosphorylation occurs at many amino acid residues including serine [75]. In the present study, many members of serine-threonine kinase family were found to be involved in the processes of peptidyl-serine phosphorylation and modification (Additional file 5).

An interesting serine-threonine kinase present was doublecortin like kinase 3 (DCLK3). DCLK3 is a DCX-domain (doublecortin) containing protein with a kinase domain attached to them. In the Apicomplexan parasites such as *Toxoplasma*, DCX loss causes impaired host-cell invasion and slow growth [76]. As with other myxozoan parasites, the role of post-transcriptional and post-translational modifications during the parasite life-cycle remains unexplored in *T. bryosalmonae*. However, the findings in this study suggest towards their potential role in regulating parasite life-cycle.

### Possible role of cysteine in mitigating oxidative stress

We determined cysteine metabolic process to be amongst the upregulated processes other than the top ten biological processes. Under this process the transcripts of CDO, glutamate-cysteine ligase catalytic subunit (GCLC) and GGT1 (65-folds, 29-folds and 3.0-foldss, respectively) were included. By involving in numerous biological pathways, these genes have been found to be important mediators of cysteine based antioxidative response [77, 78]. The upregulation of these genes in the carrier host probably indicate that cysteine metabolism may be a major oxidative stress management strategy employed by *T. bryosalmonae* in brown trout as compared to rainbow trout.

### Comparison of RNA sequencing and qRT-PCR data

Validation of RNA sequencing data by qRT-PCR is a routinely used approach. In our study, in general, a high correlation was obtained between the expression level obtained by RNA-seq data and qRT-PCR for the randomly selected transcripts. However, for one of the parasite transcripts NHP2, although both techniques revealed its downregulation in brown trout relative to rainbow trout; the difference was relatively very high. This could be merely due to the fact that both are different techniques (utilizing different normalization methods, have different dynamic range of expression detection etc.), or due to the biological complexity of the investigation. Additionally, the gene or transcript under investigation could be the potential reason [79]. This has also been observed for genes which exhibit very high or very low expression levels [80].

Overall, our study suggests that *T. bryosalmonae* regulates its gene expression depending on the host. Gene ontology enrichment analysis enabled us to identify many biological processes to which the DETs were associated such as the post-transcriptional and post-translational regulatory mechanisms, cytoskeleton organisation, stress response mediated by HSPs and cysteine metabolism, which could be important determinants of *T. bryosalmonae* fate in these two hosts. Considering the multitude of important functions that may be executed by the DETs, the proteins encoded by them should be further evaluated by functional studies as molecular targets for developing therapeutics against *T. bryosalmonae*. Additionally, functional studies will be required to determine the precise roles of identified hypothetical proteins and genes with unknown function.

### Limitations and future prospects

Though we successfully employed FACS to isolate *T. bryosalmonae* from the fish host for the first time, we acknowledge the limitations associated with our study. While we visually examined cell viability pre and post sorting and ensured the inclusion of living cells based on the forward and side scatter characteristics provided by FACS, we did not assess it using other techniques such as live and dead cell staining or the incorporation of fluorescent markers. In future, incorporating live and dead cell staining or other fluorescent markers in the FACS technique would enhance the suitability of the technique for conducting further studies as these complementary methods would enable a more precise assessment of cell viability. This technique could be pivotal in aiding the in vitro culture of this parasite.

## Supplementary Information

**Additional file 1.** **Quality control pipeline applied in the analysis of RNA-seq data.**

**Additional file 2.** **List of quantitative qRT-PCR primers used in this study.**

**Additional file 3.** **List of expression values for all *T. bryosalmonae *transcripts.**

**Additional file 4.** **List of differentially expressed transcripts of *T. bryosalmonae.***

**Additional file 5.** **List of top ten GO biological process categories of upregulated and downregulated *T. bryosalmonae *transcripts.**

## Data Availability

The datasets generated for this study are available in the NCBI Short Read Archive (SRA) portal under NCBI Bioproject ID PRJNA837187.

## Abbreviations

*T. bryosalmonae*: *Tetracapsuloides bryosalmonae*
PKD: proliferative kidney disease
DPBS: Dulbecco’s phosphate buffered saline
NCS: newborn calf serum
FACS: fluorescent activated cell sorting
RNA-seq: RNA sequencing
DETs: differentially expressed transcripts
qRT-PCR: quantitative real-time PCR
