# Supplementary figures and images for: Differentially expressed transcripts of Tetracapsuloides bryosalmonae (Cnidaria) between carrier and dead-end hosts involved in key biological processes: novel insights from a coupled approach of FACS and RNA sequencing
Source: Vet Res. 2023 Jun 26;54:51. doi: 10.1186/s13567-023-01185-7 (PMC10291810; doi:10.1186/s13567-023-01185-7)

**
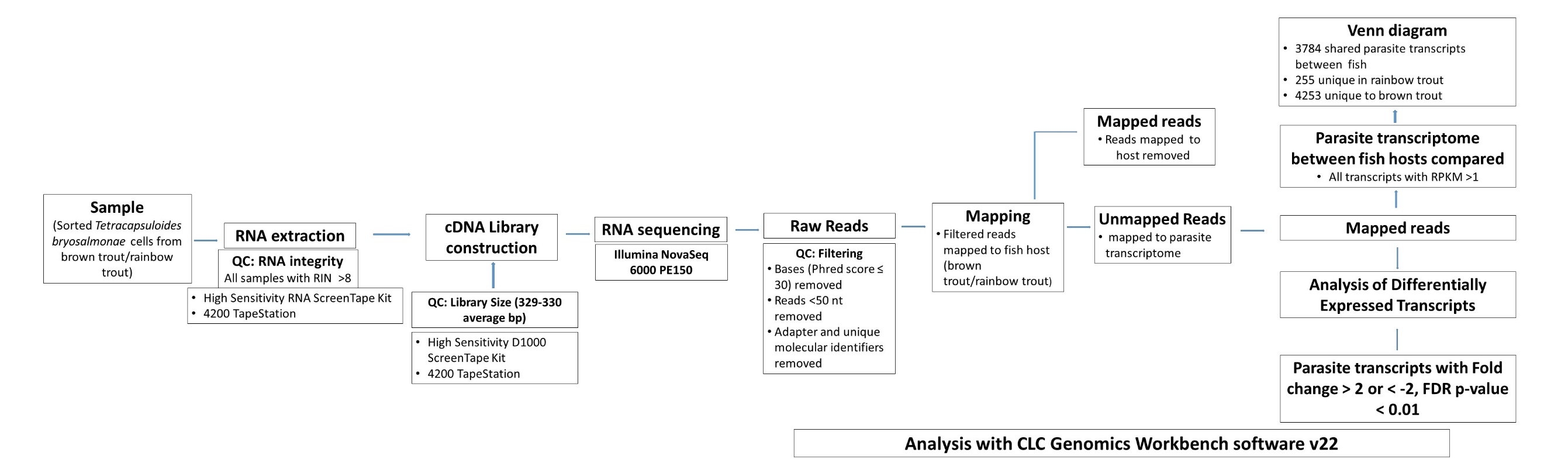
Additional file 1:** **Quality control pipeline applied in the analysis of RNA-seq data**

Supplement: Supplementary file 1 — Additional file 1. Quality control pipeline applied in the analysis of RNA-seq data. [file 13567_2023_1185_MOESM1_ESM.docx]
