# Supplementary material for: Differentially expressed transcripts of Tetracapsuloides bryosalmonae (Cnidaria) between carrier and dead-end hosts involved in key biological processes: novel insights from a coupled approach of FACS and RNA sequencing
Source: Vet Res. 2023 Jun 26;54:51. doi: 10.1186/s13567-023-01185-7 (PMC10291810; doi:10.1186/s13567-023-01185-7)
Supplement: Supplementary file 2 — Additional file 2. List of quantitative qRT-PCR primers used in this study. [file 13567_2023_1185_MOESM2_ESM.docx]

| Contig Number/  Gene Bank ID | Gene | Primer code | Sequence (5’- 3’) | Annealing Temp (°C) | Amplicon size  (bp) |
| --- | --- | --- | --- | --- | --- |
| Sac_contig_96 | ATP-binding cassette sub-family G member 4-like | ATP Bcas F | TCCATCAACCTAGTGCCAAGA | 60.0 | 187 |
|  |  | ATP Bcas R | ACTGGATGTTCATTTGTGGCT |  |  |
| Sac_contig_13766 | Gag-pol fusion protein | GagPol F | GCTGACATTTCATAAGGTTGAGC | 58.0 | 196 |
|  |  | GagPol R | ACGAACTGAGTGAAGAACAACG |  |  |
| Sac_contig_6512 | Leukocyte surface antigen CD53-like | LSA CD 53 F | AATCGTCTAGGAAAGTTATTGGTCA | 60.0 | 170 |
|  |  | LSA CD 53 R | AGGCCAGCAATGATTAGCCA |  |  |
| Sac_contig_490 | Predicted protein | PP F | ACAGCTGTAGCCCTCTTAAAGT | 60.0 | 166 |
|  |  | PP R | GGTTCCATGATTGGCTCAACT |  |  |
| Sac_contig_1694 | CD63 antigen | CD63 F | CAGGTTTAGCTACTTCTGGGT | 60.0 | 173 |
|  |  | CD63 R | ATTCCTCATCGCAGCTGTAAC |  |  |
| Sac_contig_5974 | NHP2-like protein 1 | NHP2 F | CGGAGCAGGAAATAACTGGC | 60.0 | 175 |
|  |  | NHP2 R | TTGAACCGAGGTATTGCCGA |  |  |
| Sac_contig_2707 | CWF19-like protein 2 | CWF F | TACATAGGAGCTTCGCAAATTACA | 59.5 | 182 |
|  |  | CWF R | ATGGTTGATGAGGACGTTTTTGAG |  |  |
| FR852769 | *T. bryosalmonae*  RPL18  (Faber et al. [19]) | RPL-18 F | GTAAACGGGGACAAAAAGA | 60.0 | 251 |
|  |  | RPL-18 R | GGAGCAGCACCAAAATAC |  |  |

**Additional file 2: List of quantitative qRT-PCR primers used in this study.**
